# Supplementary material for: Acetylation-Mimic Mutation of TRIM28-Lys304 to Gln Attenuates the Interaction with KRAB-Zinc-Finger Proteins and Affects Gene Expression in Leukemic K562 Cells
Source: Int J Mol Sci. 2023 Jun 6;24(12):9830. doi: 10.3390/ijms24129830 (PMC10298087; doi:10.3390/ijms24129830)
Supplement: Supplementary file 1 [file ijms-24-09830-s001.zip › Supporting information.pdf]

Supporting information

Figure S1-S4

Table S1-S5

Figure S1

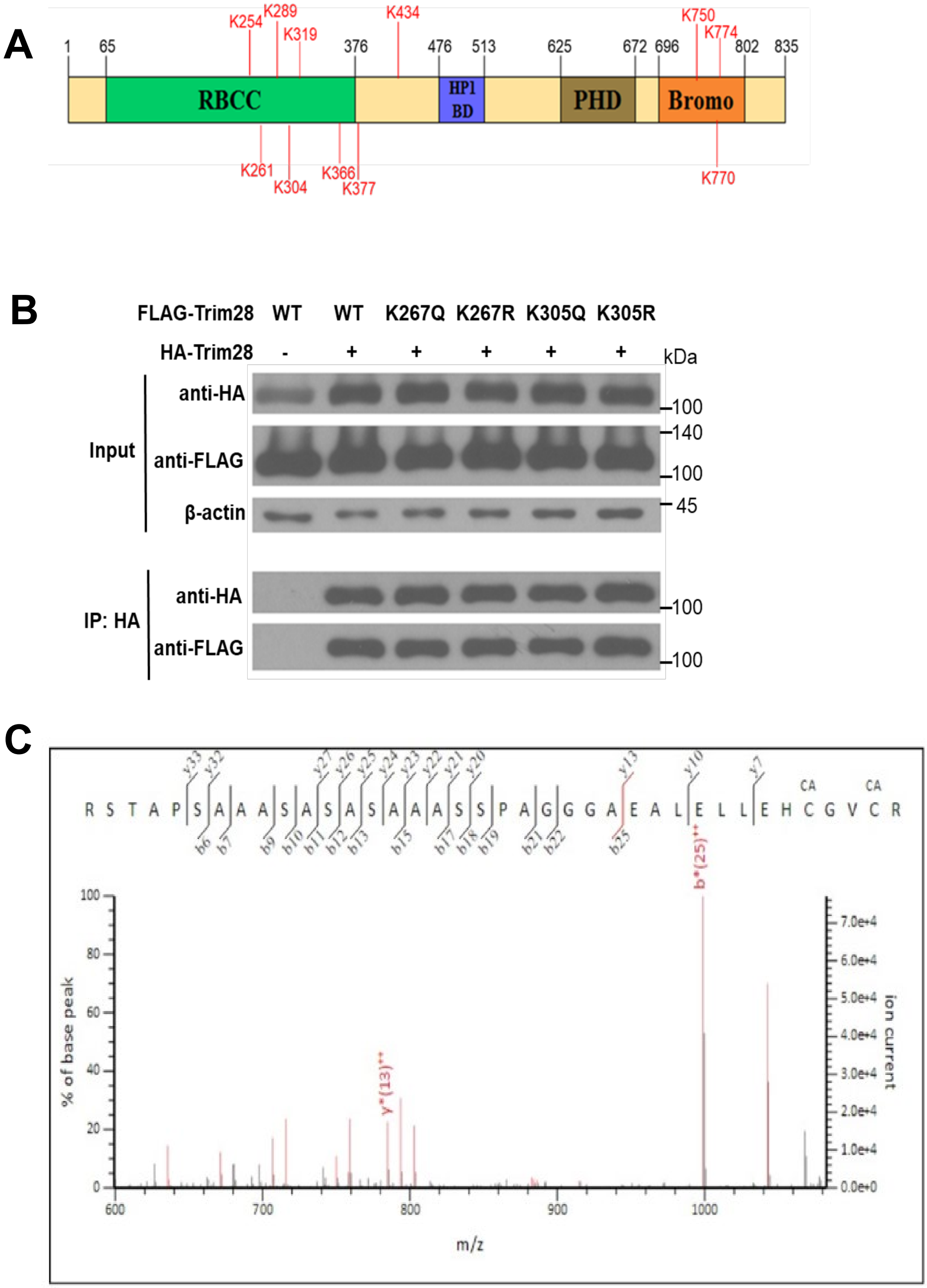

A
1
65
376
476
513
625
672
696
802
835
RBCC
HP1 BD
PHD
Bromo
K254
K289
K319
K434
K750
K774
K261
K304
K366
K377
K770
B
FLAG-Trim28
WT
WT
K267Q
K267R
K305Q
K305R
HA-Trim28
-
+
+
+
+
+
kDa
Input
anti-HA
anti-FLAG
β-actin
IP: HA
anti-HA
anti-FLAG
100
140
100
45
100
100
C
R S T A P S A A A S A S A S A A A S S P A G G A E A L E L L E H C G V C R
CA
CA
y33 y32 y27 y26 y25 y24 y23 y22 y21 y20 y13 y10 y7
b6 b7 b9 b10 b11 b12 b13 b15 b17 b18 b19 b21 b22 b25
b*(25)++
y*(13)++
% of base peak
ion current
m/z

Figure S1 Trim28 acetylation and dimerization. (A) Identification of TRIM28 acetylated residues in K562 cells. Extracts of K562 cells were immunoprecipitated with anti-TRIM28-N followed by SDS-PAGE and LC-MS/MS analysis. The detected acetylated lysines are shown in red. (B) Trim28-K305Q associates with wild-type TRIM28. Co-IP of HA-Trim28 with FLAG-tagged mouse Trim28 wild-type (WT), K267Q, K267R, K305Q, or K305R. These constructs were overexpressed in 293T cells as indicated, and protein complexes were pulled down by HA-Sepharose and detected with anti-FLAG or anti-HA. (C) MS analysis of human TRIM28. Mouse FLAG-Trim28-K305Q was overexpressed in 293T cells (human embryonic kidney) and purified by using FLAG-agarose beads for LC-MC/MC. A unique peptide from amino acids 32-69 of human TRIM28 was identified, as indicated by the sequence shown at the top.

Figure S2

A

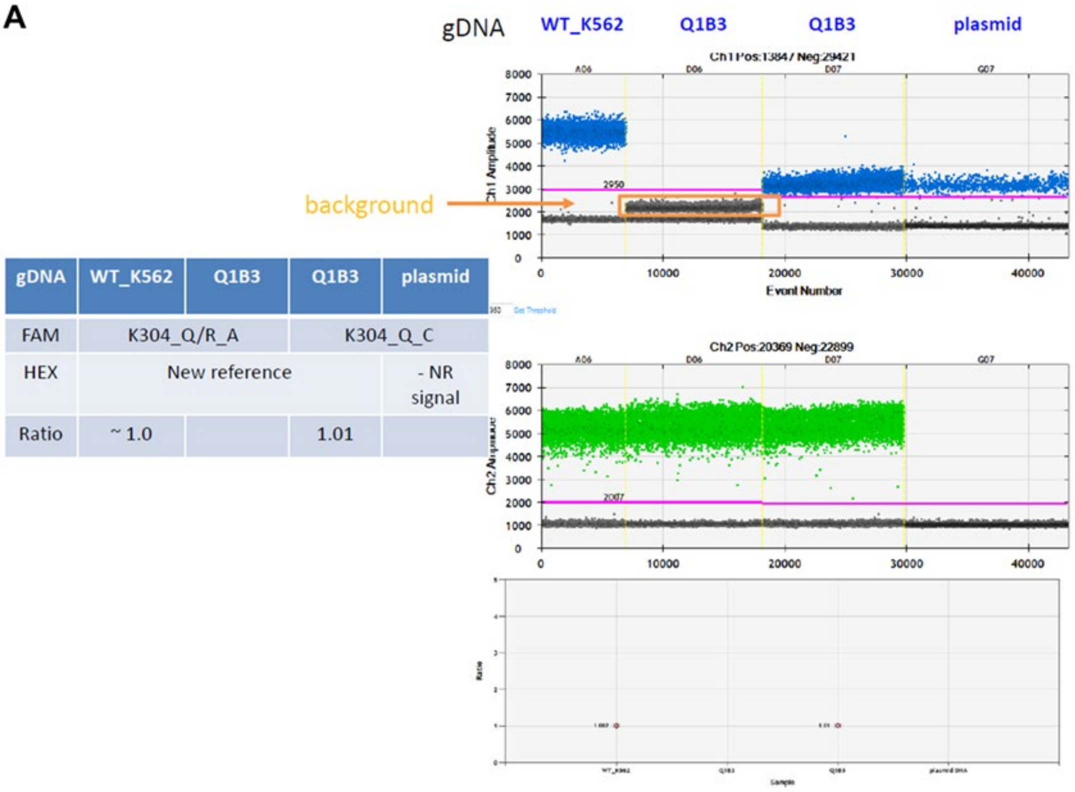

gDNA	WT_K562	Q1B3	Q1B3	plasmid
FAM	K304_Q/R_A		K304_Q_C	
HEX	New reference			- NR signal
Ratio	~ 1.0		1.01	

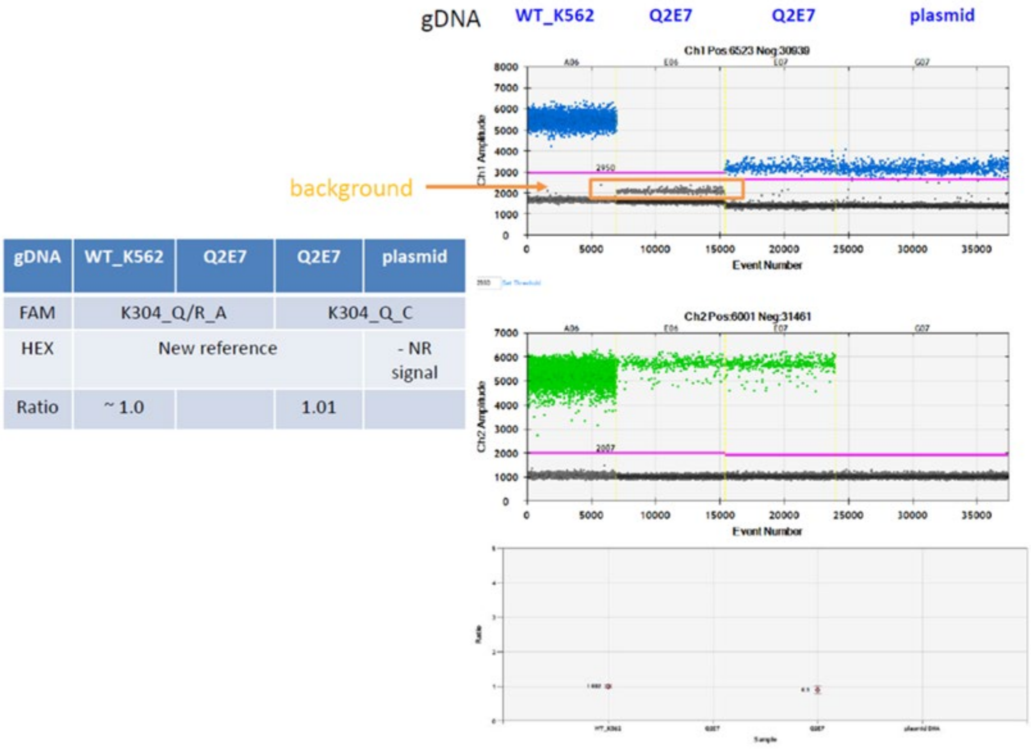

gDNA	WT_K562	Q2E7	Q2E7	plasmid
FAM	K304_Q/R_A		K304_Q_C	
HEX	New reference			- NR signal
Ratio	~ 1.0		1.01	

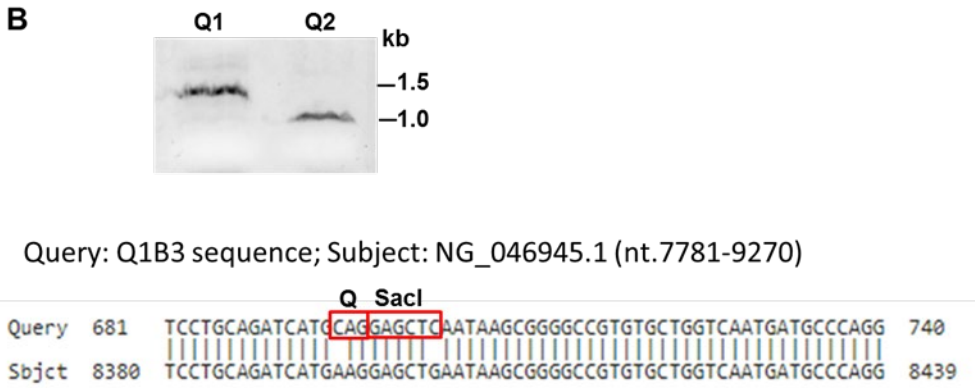

Figure S2 *TRIM28*-K304Q KI cell analysis. (A) Quantitative Droplet Digital PCR (ddPCR) confirmed knock-in. There were 50 clones subjected to SacI digestion, and 5 clones to ddPCR analysis. The FAM-labeled probes were designed to perfectly complement the wild type K304 sequence (K304_Q/R_A) or capable of binding to K304Q (K304_Q_C). The HEX-labeled probe recognized other genome sequence outside K304 region as a reference. The results showed that both Q1B3 (indicated Q1 later) and Q2E7 (indicated Q2 later) successfully K304Q knocked-in in three alleles. (B) DNA Sequencing confirmed K304Q knock-in. A 1490 bp region of TRIM28, containing the Cas9-RNP targeting site, were PCR amplified by using the TRIM28 gDNA primer set (forward 5'– 7781 CTCTACATCTTCCCAATAAATGGCCCAGTG – 3', and reverse 5'– 9270 TGTGAACAAAGCAGAACCCTCTGCCTCAGT – 3'). The PCR fragments from Q1 or Q2 clone were separated on agarose gel and ligated into TA vector for Sanger sequencing. Q1B3 displayed correct size from 7780 to 9270 and mutated sequences (K304Q and SacI site) but Q2E7 was incorrect size from 7780 to 8550.

Figure S3

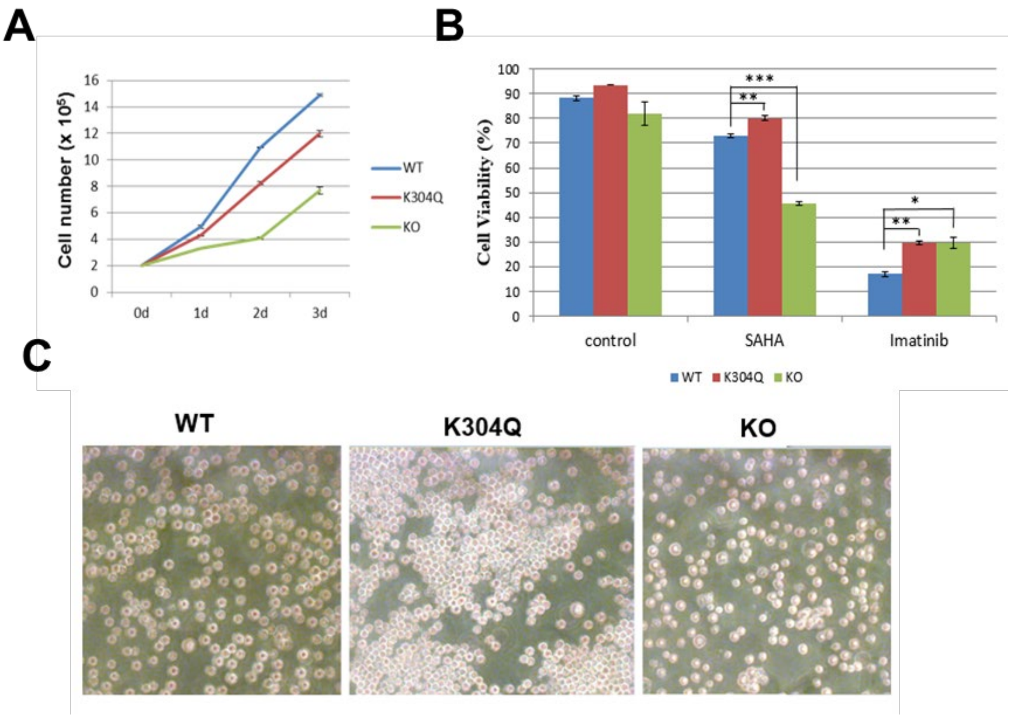

Figure S3. Comparison of phenotypes between wild-type, *TRIM28*-K304Q KI and *TRIM28*- KO K562 cells. (A) Cell proliferation analysis. Wild-type (WT), *TRIM28*-K304Q and *TRIM28*-KO K562 cells were seeded as $2x10^5$/ml and cultured for 1d, 2d, and 3d. The cell number was determined with trypan blue stain and calculated with automatic cell counter. (B) Drug sensitivity analysis. The cells were treated with a HDAC inhibitors SAHA (2 μM) or Imatinib (2 μM) as indicated for 72 h and cell viability were analyzed by trypan blue staining and automatic cell counter. (C) *TRIM28*-K304Q cells were more attachable than wild-type and KO cells.

Figure S4

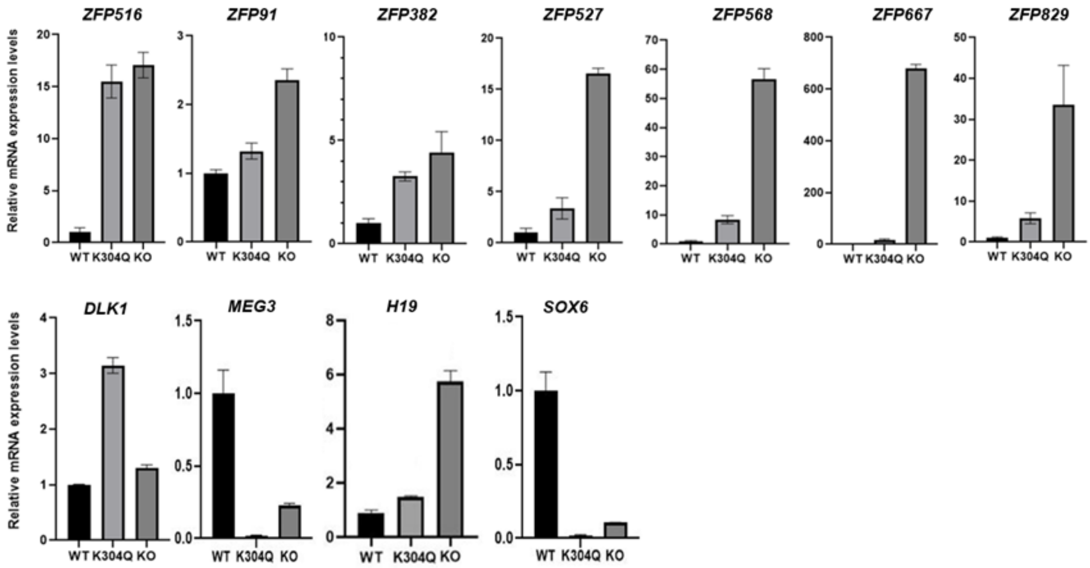

Figure S4 RNA-seq verification. RT-qPCR assays verified the identity of certain ZNFs, imprinting genes, and *SOX6* in WT, *TRIM28*-K304Q KI and *TRIM28*-KO cells.

Table S1

The differential gene expression between wild-type and

***TRIM28*-K304Q KI cells (excel file)**

Table S2A and S2B

IP-MS analysis of the associated proteins of wild-type- and K304Q-TRIM28 (excel files)

Table S3A Proteins specifically interacted with wild-type TRIM28

Epigenetic regulators	EHMT1, EHMT2
Protein ubiquitination	CTLH complex: RANBP9, WDA26, GID8, MAEA, RBP10, ARMC8, RMD5A, RMD5B, MKLN1, YPEL5, GID4<br>MAGE family: MAGEC2, MAGEA9<br>UBP2L
RNA modification	YTHD2, ZCCHC4, FBRL
RNA degradation	NEXT(nuclear exosome target): MTREX, (ZCCHC8: K304Q), RBM7<br>Decaaping : EDC4
Translation regulation	GCN1,
Metabolism	G3PD

Table S3B Proteins interacted with wild-type and K304Q TRIM28

Heterochromatin proteins	CBX5, CBX3, CBX1<br>CENPV (centromere protein V)
Histones	H4, H2B2F, H2B1B, (H3.1 and H2A1B:K304Q)<br>(H2A1D, H2AV and H3.3: WT)
RNA binding and processing proteins	hnRNP M, hnRNP H, hnRNP F<br>PABP1, PABP4<br>REXO5 (RNA exonuclease 5)<br>DHX9
Transcription factor	GATA1
Iron metabolism	HBE, TFR1 (Transferrin receptor protein 1)

Table S4

The phosphorylated residues in wild-type- and K304Q-TRIM28

WT	K304Q	
phosphorylated residues	phosphorylated residues	peptide sequence
S19	S19	AASAAAASAAAASAASGSPGPGEGSAGGEKR
S50,S51	S50, S51	RSTAPSAAASASASAAASSPAGGGAEALELLEHCGVCR
T113		LLPCLHSACSACLGPAAPAAANSSGDGGAAGDGTVVDCPVCK
S138		DIVENYFMRDSGSK
S258	S258	LLASLVK
S350	S350	FASWALESDNNTALLLSK
S437, S439, S440	S437, S440	QGSGSSQPMEVQEGYGFGSGDDPYSSAEPHVSGVK
S473	S473	SRSGEGEVSGLMR
S489, S501	S489, S501	VSLERLDLDLTADSQPPVFK
T531, T541	T531, T536, T541	GAAAAATGQPGTAPAGTPGAPPLAGMAIVK
S594, S596, S598,T599, S600, S601, T611, S612	S594, T599, S600, S601, S612	LASPSGSTSSGLEVVAPEGTSAPGGGPGTLDDSATICR
S681, S683	S681, S683	EEDGSLSLDGADSTGVVAK
S697	S697	EEDGSLSLDGADSTGVVAKLSPANQR
S752,S756, S757	S752,S756, S757	LQEKLSPPYSSPQEFAQDVGR
S824, S828	S824, S828	FSAVLVEPPPMSLPGAGLSSQELSGGPGDGP

The red residues indicate phosphorylation in both wild-type and K304Q TRIM28; the green residues indicate phosphorylation only in wild-type TRIM28. The T536 phosphorylation only detected in TRIM28-K304Q, showed in blue type.

Table S5 Primers for site-directed mutagenesis and qPCR

A. Primers for mouse Trim28 site-directed mutagenesis		
Name	Forward (5'-3')	Reverse (5'-3')
K255Q	GAACCAACGTCAACTCTTGGCTTC	CTCACTGCATCTTCCAAAAAC
K255R	GAACCAACGTAGACTCTTGGC TTC	CTCACTGCATCTTCCAAAAAC
K267Q	GTCTTGGGGACCAACATGCCACAC	GTTTCACCAGTGAAGCCAAG
K267R	GTCTTGGGGACAGACATGCCACAC	GTTTCACCAGTGAAGCCAAG
K290Q	CTGATGTGCAGCAGCGAGTGCAGG	ACACCTGGCGGATCGAGCTTC
K290R	CTGATGTGCAGAGGCGAGTGCAGG	ACACCTGGCGGATCGAGCTTC
K305Q	CTGCAGATCATGCAGGAGCTGAATAA	AATGGCCATCTTGACATCAAC
K305R	CTGCAGATCATGAGGGAGCTGAATAA	AATGGCCATCTTGACATCAAC
K341Q	CAAAATTCAGAGGCACCAGGAAC	GACATGGTCCAGTGCTGGCG
K341R	CAAAATTCAGCAGCACCAGGAAC	GACATGGTCCAGTGCTGGCG
R310Q	CTGAATAAGCAGGGTCGAGTTCTG	CTCCTTCATGATCTGCAG
Primers for human TRIM28 site-directed mutagenesis		
Name	Forward (5'-3')	Reverse (5'-3')
K304Q	GCAGATCATGCAGGAGCTGAATAAG	AGGATGGCCATCTTGACA
K304R	GCAGATCATGAGGGAGCTGAATAAG	AGGATGGCCATCTTGACA

B. qPCR primer sequences		
Gene	Forward (5'-3')	Reverse (5'-3')
ZNF516	GCACACTCAGTGGTGTTTGAG	GGACATCGTGAGGGTACTGC
ZNF667	TGTGACAAGTTCTTCAGGCG	GGATGAATGCCGATTGCAGAC
ZNF382	CCTGCTCAGAAGGCGCTTTACA	CTCTGTGTCCATAGCTCTTCTCC
ZNF568	AAGAGTCTGCCCTTTCCGAGGA	GCAGGTTTCATTTGCTCCCACTC
ZNF829	ATGGGAATGCCTGGACGCTGAT	CCAGGGCTCTTTTCCTTGTTCC
ZNF527	GAGTGGGAATGGCTGAAGCCAT	CCAGTAAGGAGATCATGTTGGGC
ZNF91	AGGAGTGGCAATGTCTGGACAC	CAGGGCTCTTTTCCTTGCTCCA
ZNF445	AGCTCCAGGAGACCATGACT	GAATGGTCCCACCAGGGAAG
H19	TGCTGCACTTTACAACCACTG	ATGGTGTCTTTGATGTTGGGC
IGF2	GGGCAAGTTCTTCCAATATGA	TCACTTCCGATTGCTGGC
PEG3	CGGAACAGAAGAGAGTCCTCAC	TGCTTCTTGGGTTCCTGGTGTG
MEG3	TTTTGTGCCCAAGGCTCCTGGA	AGGGACTCAAGGAGCCAGGTTA
DLK1	GCACTGTGGGTATCGTCTTCC	CTCCCCGCTGTTGTACTGAA
ITGB3	AGAGCCAGAGTGTCCCAAG	GGCCTCTTTATACAGTGGGTTGT
HBG(1/2)	TGGATGATCTCAAGGGCAC	TCAGTGGTATCTGGAGGACA
HBE1	GCAAGAAGGTGCTGACTTCC	ACCATCACGTTACCCAGGAG
SOX6	CGGTCTACCTACTGGGATAA	GCTTTTGTTTGGCAGATTGA
IKZF2	ACACTCTGGAGAGAAGCCGTTC	CCAGTGAACTGCGCTGCTTGTA
TRIM28	AAGGACCATACTGTGCGCTCTAC	ACGTTGCAATAGACAGTACGTTCAC
GAPDH	CAACAGCGACACCCACTCCT	CACCCTGTTGCTGTAGCCAAA
ACTIN	GCACCAGGGCGTGATGG	GCCTCGGTCAGCAGCA

C. ChIP primer sequences		
Name	Forward (5'-3')	Reverse (5'-3')
H19 promoter	CACGCTCAGGGATCATCACG	TGTGGGCAAATTCACCTCTCC
LINE1 promoter	GAACGCCACAAAGATACTCC	CTCTTCTGGCTTGTAGGGTTTCTG
LCR-HS3	ATAGACCATGAGTAGAGGGCAGAC	TGATCCTGAAAACATAGGAGTCAA
ZNF568 3'exon	GCAACAGGAAACACTTGTGAGG	AGGGGGTTACAACATAGGATGC
ZNF667 3'exon	AGTTCTTCAGGCGGCTTTCA	ATGCCGATTGCAGACCTTCT
ITGB3 promoter	CCTATCACTGCTTACGCAAGC	CCGGTAGACTACCTACCTGTT
